# Association between the attentional network efficiency and change of direction speed ability in young male Indian footballers

**DOI:** 10.3389/fspor.2025.1529252

**Published:** 2025-01-29

**Authors:** Debabrata Chatterjee, Santi Ranjan Dasgupta, Arkadeb Dutta

**Affiliations:** ^1^ Neuro-Cognition Laboratory, Department of Sports Science and Yoga, Ramakrishna Mission Vivekananda Educational and Research Institute (RKMVERI), Belur Math, India; ^2^ West Bengal State Council of Sports, Department of Youth Services and Sports, Govt. of West Bengal, Kolkata, India; ^3^ Department of Sports Medicine, East Bengal Football Club, Maidan Tent, Kolkata, India; ^4^ Department of Biomedical Science & Technology, School of Biological Sciences, Ramakrishna Mission Vivekananda Educational and Research Institute (RKMVERI), Narendrapur, India

**Keywords:** CODS, football, attentional networks, executive control, cognition

## Abstract

**Introduction:**

Interactions between cognitive functions and sports-specific motor actions are crucial for strategic sports performance. Change of direction speed (CODS) is an essential motor ability required for rapid positional maneuvering in football. Although CODS lacks perceptual judgment and anticipatory elements of higher-level cognition, its connection with fundamental cognitive abilities cannot be undermined. The attentional networks is the basis of the fundamental cognitive abilities controlling complex behavior. The present study aimed to investigate the association between CODS ability and the efficiency of alerting, orienting, and executive components of the attentional networks, and decision-making in footballers.

**Methods:**

Seventy-eight male footballers (age: 15.4 ± 0.87 years, BMI: 19.4 ± 1.98 kg/m^2^) during pre-season completed a battery of field tests comprising Illinois agility test (IAT), 30 m sprint, standing broad jump, and Yo-Yo test. Attentional network components and decision-making ability were tested in the participants with computerized Attentional Network Test-Interactions (ANT-I) and choice reaction time (CRT) tasks in the laboratory set-up. A 2(alerting) ×3 (orienting) ×2 (executive) repeated measures ANOVA tested interactions between the attentional network components. Partial correlation was conducted between the physical (field tests) and cognitive test scores adjusted for age and BMI.

**Results:**

CODS ability measured with IAT was significantly correlated [*r* = +0.507 (large), *p* < 0.05] with the executive control network only, nor with alerting [*r* = −0.039 (trivial), *p* > 0.05] and orienting [*r* = + 0.051 (trivial), *p* > 0.05] networks and neither the CRT task performance [*r* = −0.011 (trivial), *p* > 0.05].

**Discussion:**

A strong positive association between executive control and preplanned CODS indicates better interference control by the attentional network. The later may be a factor for faster CODS execution in young footballers. Hence, it may be concluded that better CODS ability is possibly an outcome of innate competence in executive control of the attentional network in young male footballers. These findings attempted to fill the knowledge gap by highlighting the importance of the attentional network functions in modulating CODS ability. The outcomes can benefit football training by implementing ANT-I test in sports-specific settings and for screening purposes. However in the future, a large-scale study including female footballers is required to strengthen this claim further.

## 1 Introduction

Cognitive abilities at optimal levels are essential for superior performance in the athletes of open-skill, strategic sports (1, 2). The higher-order cognitive elements such as visual scanning, judgment, planning, and decision-making are required to process relevant information in the dynamic, externally-paced environment to provide appropriate behavioral responses in the shortest possible time (3–5). Executive functioning and attentional control are the fundamental cognitive abilities, which are the basis of higher-order cognitive processes implemented in sports-specific environments (1, 6). The executive functions have three core mental disciplines that monitor and continuously update the information of a situation, switching to relevant stimuli in the environment and selectively allocating attention to them by restricting interference from irrelevant thoughts of action (7). Therefore, it becomes the source of goal-directed behavior.

Football is an open-skill, strategic sport characterized by short bouts of intermittent sprints, rapid change of direction and velocity, and agility (8, 9). There are several evidences showing that executive functioning is comparatively better in expert footballers than in low-level players and novices (10–13). Expert players are also efficient in integrating higher-order cognitive capacities with motor coordination in game-specific environments for faster and more accurate anticipatory response (2). These complex cognitive skills are regulated by the basic executive functions indicating that athletes must depend on their executive functions to excel in performance (6, 7).

Cognitive and motor skills in sports-specific settings are mutually related (14). Hence, it is becoming increasingly important to identify these cognitive functions involved in sports-specific motor behavior in football (14, 15). This approach can assist in recognizing talents based on the processing efficiency of brain networks regulating these cognitive attributes. Moreover, a detailed understanding about the interaction between these brain networks and motor abilities can help to improve sports-specific actions with standard drills or dual-task training program (16).

CODS defined as “the ability to decelerate, reverse or change movement direction and accelerate again”, is an essential motor skill frequently performed by footballers during competitive games for gaining, holding ball possession evading opponent players, and during off-the ball situations (17, 18). CODS ability is a critical parameter for elevating a player's performance along with endurance, speed, and explosive strength (19, 20). In a football match, CODS occur in every 2–4 s regardless of playing positions of the footballers (21). Footballers can perform 726 ± 203 changes in CODS during a match, and with 609 ± 193 turns in completely opposite direction or 90° to the left or right (22). CODS is also an important attribute for grouping adolescent sub-elite players with cluster analysis (23). Hence, CODS ability is crucial for on-field performance prediction (24), and for identifying elite performers in football (25). Several standardized CODS drills are recommended by coaches and sports science experts for the long-term development of motor skills and abilities in football (20). However, in real game scenario the change of direction executed most of the time is a reactive response to unpredictable stimuli and therefore varies from the standard CODS tests (26). Standard CODS ability is a closed-skill technique and devoid of anticipatory action (26). Due to the multiple changes in direction with high speed in a preplanned course, standard CODS ability tests lack higher-level brain processing for perceptual judgment and reactive decision-making (26).

For this reason, CODS training is now believed to be ecologically less valid relative to agility drills for adapting players to situations mimicking match scenarios where there is a demand for reactive response (27). However, it would be premature to draw an inference that there are no possible links between CODS ability and other cognitive disciplines. A qualitative evaluation of externally focused attention on CODS was promising (28). A recent study showed that footballers trained with cognitive-motor dual-task developed better CODS ability (29). These findings have provided hints about the involvement of cognitive abilities in preplanned CODS or when navigating in the sports-specific environment in a football match (16, 29). However, the nature of basic cognitive components and their regulatory brain networks in controlling CODS are unknown. In this context, the present study has explored a possible intersection of the attentional networks with CODS ability in young footballers. This is because the attentional networks form the basis of the fundamental cognitive abilities and span across diverse brain regions regulating attention and executive functioning (30). The findings can improve our limited knowledge about the importance of attentional control and executive functions in CODS behavior. This can have significant implications for sports psychology and athlete development.

It must be mentioned that there are difficulties in designing paradigms to measure core executive functioning during on-field CODS ability or other domain-specific tasks (2). An alternative approach, reported earlier for examining executive functioning in physical activity and sports, can be by using standard laboratory-based computerized tasks of executive functioning in athletes and associating with the on-field CODS ability measures (11).

For this purpose, a conventional computerized task of assessing the attentional networks can be used. The attentional network comprises three separate functional components, namely alertness, orienting, and executive control (31). Each of these components runs at three anatomically distinct brain regions that are interconnected. The alerting component can be intrinsic or phasic and seats at the locus coeruleus and the parietal and right frontal cortex (31). The orienting component can be reflexive or goal-directed, localized in the frontal eye fields, the superior colliculus, the temporal parietal junction, and the superior parietal cortex (31). The executive component consists of complex mental operations for monitoring and interference control using the basal ganglia, anterior cingulate, and lateral ventral prefrontal cortex (32). Each of these components and their interaction is crucial since the functional deficit of networks can cause serious cognitive and neuropsychological health concerns (33). Earlier a study showed that generally athletes from strategic sports exhibit better attentional network functioning (34). However, the contribution of the individual network components on CODS ability and other physical attributes of football is not well known.

Hence, we hypothesize that footballers with greater efficiency in the attentional networks are better performers in CODS-ability performance measures. To test this hypothesis, we aim to investigate the association between CODS ability with each of the attentional network components using the attentional network test- interactions (ANT-I) task at the intra-individual level in young male professional footballers.

## 2 Materials and Methods

### 2.1 Study design

A cross-sectional design on young male football players was implemented to explore the associations between the cognitive parameters with the physical attributes namely, agility, lower limb explosive power, speed, and endurance ability. Eligibility criteria were male footballers from the same socioeconomic status and with self-reported normal vision and no neurological deficits.

### 2.2 Subjects

Eighty-eight, young, male football players affiliated with an elite football academy (accredited by the All India Football Federation) were registered for this study. The weekly schedule of all the participants comprised five field-based and one gym-based training session, a maximum of one practice match. Participants had at least three years of playing experience in football. Ten athletes were excluded due to lack of continuity or injury in the middle of the study. Finally, the tests were conducted on 78 athletes having an age range between 14 and 17 years (age: 15.4 ± 0.87 years, height: 167.2 ± 5.86 cm, body mass: 54.4 ± 6.58 kg; BMI: 19.4 ± 1.98 kg/m^2^), including 9 goalkeepers, 17 defenders, 20 defensive midfielders, 19 attacking midfielders, and 13 forwards. We selected these age groups because attentional network components become developed and reaches adulthood stability (35). The age groups are also suitable to identify and nurture future elite athletes (36). At the time of conducting the test battery, all players were injury-free. The tests were executed in two alternate days. All the tests were conducted during the preparatory phase in the academy. The on-field testing sessions were performed between 9 and 11 am to avoid diurnal variation. At the beginning of the testing, footballers performed a routine warm-up for approximately 10 min which included a general warm-up, dynamic stretching, and specific warm-up exercises. An active rest of 3–5 min was provided before the testing. The protocol followed the guidelines of the Declaration of Helsinki and was approved by the Institutional Ethics Committee (IEC/2023/07/SSY01). Prior to the commencement of the study, participants and their parents were informed about the purpose, benefits, and risks of the investigation. Written consents signed by the legal guardians of the participants were collected.

### 2.3 Sample size estimation

*A priori* sample size estimation for all the correlation analysis was conducted under a bivariate normal model using G*POWER 3.1.9.7 (37). The minimal sample size was estimated to be 67 at the 0.05 alpha level with the power set at 0.8, and using Cohen's medium effect size of 0.3 (38). With a 10% dropout estimation, the total sample size required was at least 74. For repeated measures within-factors ANOVA used in the attentional network interaction task, a medium effect size of 0.25 was considered (38). The estimated total sample size was twenty with the number of groups and measures set as two and six respectively.

### 2.4 Procedures

#### 2.4.1 Cognitive measures

The computerized cognitive tests used were an ANT-I task and a CRT task, designed and presented in Psychtoolbox 3 (39) using custom-written Matlab codes (Matlab 2018a version software).

##### 2.4.1.1 Attentional network interaction task (ANT-I)

ANT-I, a modified version of the Attentional Network Task was used for measuring attentional network indices (30, 40). Previous literature indicated ANT-I as a convenient measure to assess attentional networks with high reliability (41). ANT-I can assess higher-order complex cognitive actions and has an advantage over other tests that measure the processing speed of a simple reaction time in sports (30, 42).

ANT-I was conducted in a dimly lit room devoid of distracting noise. Participants were seated 60 cm away from a 15-inch color monitor used for displaying the ANT-I task stimuli. Before recording, participants were instructed about the task and the given practice session where they had to respond quickly and correctly by pressing the “C” (left) or “M” (right) keys on a standard computer QWERTY keyboard, based on the direction of the central arrow (0.55° long pointing either left or right) flanked by two other irrelevant but identical arrows on either side (0.06° away from each other).

**Figure 1:**
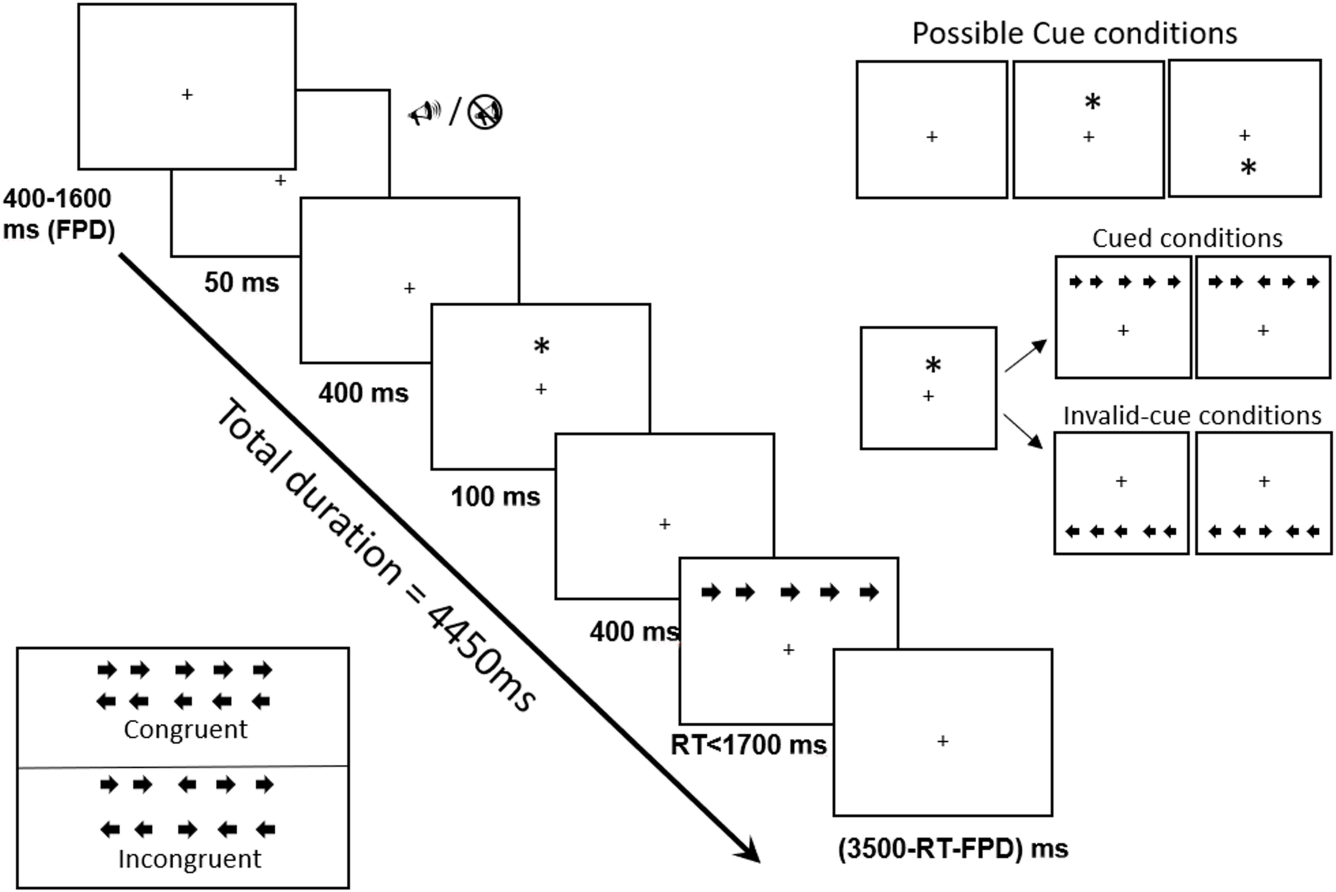
Schematic illustration of the attentional network test—interactions (ANT-I). The direction of the diagonal arrow in bold represents the sequence in a trial starting with variable fixation point duration (FP or FPD) between 400 and 1,600 ms, followed by any of the two alerting state (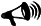: tone; 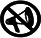; no tone); then any three orienting conditions (no-cue, cue, invalid-cue) [see illustration at the top right corner]; and finally the reaction time window (RT) with two congruency levels [see illustration at bottom left corner for 4 possible combinations of congruent and incongruent flankers]. Cue and invalid cue is provide with “*”.

The task began with a set of trials and each trial consisted of sets of events (Figure 1). Each trial began with the appearance of a cross-hair (“+”) for variable duration between 400 and 1,600 ms. Participants were encouraged strongly to fix gaze on the central crosshair. This was followed by a presentation of a 2 KHz alerting tone for 50 ms duration in half of the trials. After a 450 ms stimulus onset asynchrony (SOA), a visual orienting cue in the form of an asterisk (“*”) was presented above or below the fixation point for 100 ms in two-thirds of the trials. After another 500 ms SOA, the target arrow with flankers was displayed in identical or opposite locations of the cue until a response was made or for a maximum of 1,700 ms. After the response, the fixation point was shown for a variable duration adjusted from the participant's RT and the fixation time at the beginning of the trial so that every trial duration was maintained constant at 4,450 ms. Participants first completed a practice block, followed by three experimental blocks of 48 trials each without feedback, with approximately 1-min breaks in between. The ANT-I session lasted around 15 min.

Each block had two alerting levels with half with alerting tone and the remaining half with no tone (alerting vs. non-alerting); three cued conditions with cue matching target location (cued), cue opposite to target location (uncued invalid cue) and no-cue trials; two congruency levels with congruent trials (50% of trials) with four flanking pointing in the same direction as the target arrow, and incongruent trials (the remaining 50%) featured flankers pointing in the opposite direction. The congruency level designates the executive control network of attention. Cued location and alerting levels define the orienting and alerting networks of attention respectively. The network scores and their interactions are assessed by recording response accuracy (i.e., percentage of errors) and reaction times (RTs). According to previous literature, RT ranging between 200 ms and 1,200 ms were considered for analysis of the three network scores (41). Previous ANT-I studies in normal subjects and young male footballers reported the attentional network scores usually in the range of 24–72 ms (30, 41, 43).

##### 2.4.1.2 Choice reaction task (CRT)

Participants were seated about 60 cm from a 15-inch color monitor in a dimly lit, noise-free room. They were instructed to focus on a crosshair (“+”) in the center of the screen with their right index finger placed on the “M” key, and the left index finger placed on the “C” key. Two boxes, one present to the left and the other to the right of the “+” and an “X” may appear in either box randomly but with equal probability in a total of sixty trials. Participant had to press the “M” key as quickly as possible with the right finger if the “X” appears in the right box or press the “C” key with the left finger if the “X” appears in the left box. Twenty trials were given as practice before the test. Inter-trial interval was set at 500 ms, with a minimum of 100 ms and a maximum of 1,500 ms allowed for motor response. The total procedure lasted for about 5 min. RT and number of correct responses were recorded.

#### 2.4.2 Physical tests

##### 2.4.2.1 CODS

**Figure 2:**
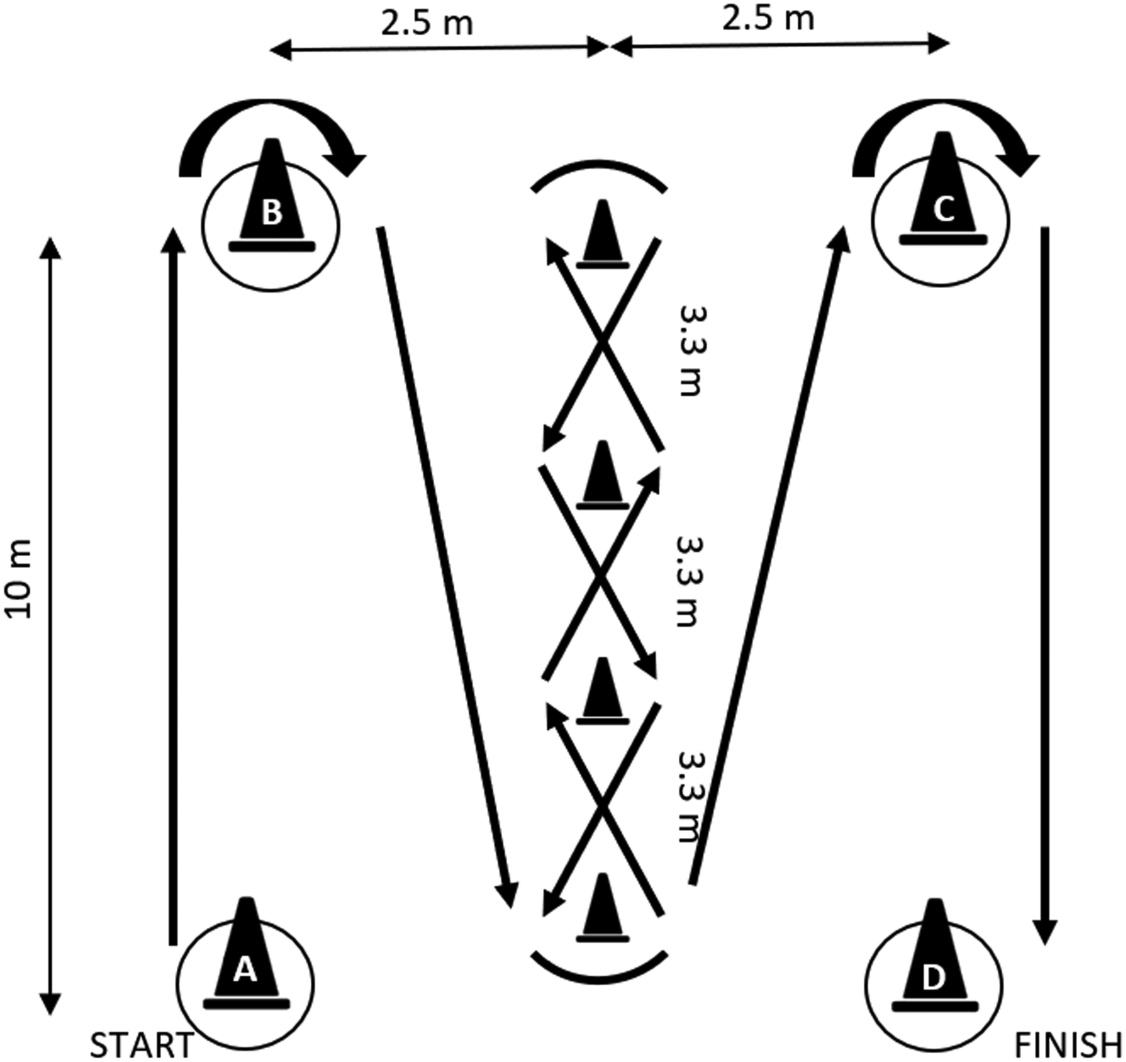
Schematic representation of the Illinois agility test. The test is set up with four cones forming 10 m by 5 m area. Cone A, marks start; Cones B and C mark the turning spots; Cone D marks the finish; the arrows in bold marks the route and direction of the pre-planned path.

The Illinois Agility Test (IAT) generally is a highly reliable and valid test to measure the CODS ability in athletes (44). Previously, IAT tested in young soccer players is now considered as a standard measure for quantifying CODS ability for high reliability (17). In the test, four cones were positioned to denote the starting point, two turning points, and the finishing point. Additionally, four more cones were arranged in the center of the course at equal intervals (3.3 m) (45). Participants were in a prone position at the starting cone and initiated the run following the “Go” command. The IAT path comprising multidirectional movements is illustrated in Figure 2. The trial is complete when the athlete crosses the finish line and no cones are knocked over. The total time taken from start to finish was recorded using an electronic hand-held timer by an experienced tester with earlier established reliability (46). The reported range of completion time of IAT in male young footballers is usually between 16 and 21 s (17, 44).

##### 2.4.2.2 Endurance

Yo-Yo Intermittent Recovery Test 2 (YYIR2) was conducted to assess maximal aerobic capacity (VO_2_max) indirectly on-field. The test is advantageous as it is simple, cost-effective, reliable, and can be administered to multiple players simultaneously (47). The test utilizes high-intensity intermittent bouts of exercise and thus meets the specific demands of football (48). Briefly, the YYIR2 consists of repeated 40 m (2 × 20 m) runs between markers set 20 m apart, which have to be performed at progressively increasing speeds dictated by an audio signal (48). Athletes also performed 10 s of active recovery between each running bout, consisting of a 10 m (2 × 5 m) walk. The test was terminated if the player failed to reach the end line within the given time frame on two consecutive occasions or reached volitional exhaustion. The maximal distances reached, counted by total laps completed, were used to estimate VO_2_max using a standard equation (47). The physiological response to YYIR2 test recorded previously in male professional footballers were in the range of 43.2–57.2 ml/kg/min (48).

##### 2.4.2.3 Explosive strength

Lower limb explosive power is an important physical factor in football. Standing broad jump (SBJ) is high reliable field-test and is commonly used to measure lower limb explosive power in youth (49). SBJ Starting from an erect position with feet placed parallel and knees bent at nearly a 90° angle, the athlete executed SBJ. All athletes were instructed to jump as far as possible and land on both feet without falling backward. The straight-line distance was measured from the starting line to the back of the heel or the closest body part to the start line after the jump with a measuring tape (50). The distance was recorded to the nearest centimeter. Each participant performed a total of three trials, among which the longest jump was used for analysis. A minimum of 2 min of rest was given between each trial. In young male footballers, SBJ ranges 1.7–2.25 m (51).

##### 2.4.2.4 Speed

Speed, reflected in sprinting ability, is one of the crucial performance measures in professional football (52). A 30 m sprint test was conducted to assess sprinting ability. Athletes continued to run at maximum speed following the “Go” command on a 30 m straight track. The run time was recorded using a stopwatch. The minimal time out of three trials was used for data analysis. The participants were given a 3-min rest break in between trials sufficient for a full recovery. A previous study on 30 m sprint in professional footballers reported an average completion time of about 4.2 s (53).

### 2.5 Statistical analyses

Data analysis was carried out in Matlab version 2018a and statistical analyses were conducted using IBM SPSS Statistics v27 Software (SPSS Inc., Chicago, IL). The mean RT (ms) and error rate of each participant across all of the 12 combinations of the ANT-I task were calculated. The mean of the CRT and physical test parameters across the participants were also calculated. Normality was checked using Kolmogorov-Smirnov test. A 2 (alerting: tone/no tone) ×3 (orienting: valid/invalid/no cue) ×2 (executive control: congruent/incongruent) within-factor repeated measures ANOVA was performed in normally distributed RT from the correct trials (30). Where necessary the Greenhouse-Geisser correction was applied to adjust the lack of sphericity in the repeated measures ANOVA. The Bonferroni confidence interval adjustment for main effects and simple main effects for interaction between the factors were performed. To understand the magnitude of differences, the effect size was calculated as partial ETA-squared (*η*_p_^2^) with the magnitude of 0.01 considered as small, 0.06 and 0.14 as medium and large respectively (54). Attentional network scores for alertness, orienting, and execution were also calculated for correlational analysis according to a previously established method (41). The alertness network score was calculated by subtracting the RT of tone from the no-tone condition. The orienting network score was obtained by subtracting the RT of a valid cue from an invalid cue. The executive control network score was obtained by subtracting the RT of the congruent condition from the incongruent condition. Higher scores in alerting and orienting indicate better efficiency in these networks. Whereas, the executive network is efficient if its network score is low (55). Pearson's partial correlation was used to assess the associations between the cognitive and physical variables with adjusted age and BMI (if any). Spearman's partial correlation coefficient was calculated if found to be not normally distributed. The magnitude of the correlation coefficient was considered small (0.1 ≤ *r* < 0.3), moderate (0.3 ≤ *r* < 0.5), large (0.5 ≤ *r* < 0.7), very large (0.7 ≤ *r* < 0.9) and nearly perfect (*r* ≥ 0.9). An alpha level of *p* ≤ 0.05 was used to determine statistical significance.

## 3 Results

### 3.1 Attentional network functioning

**Figure 3:**
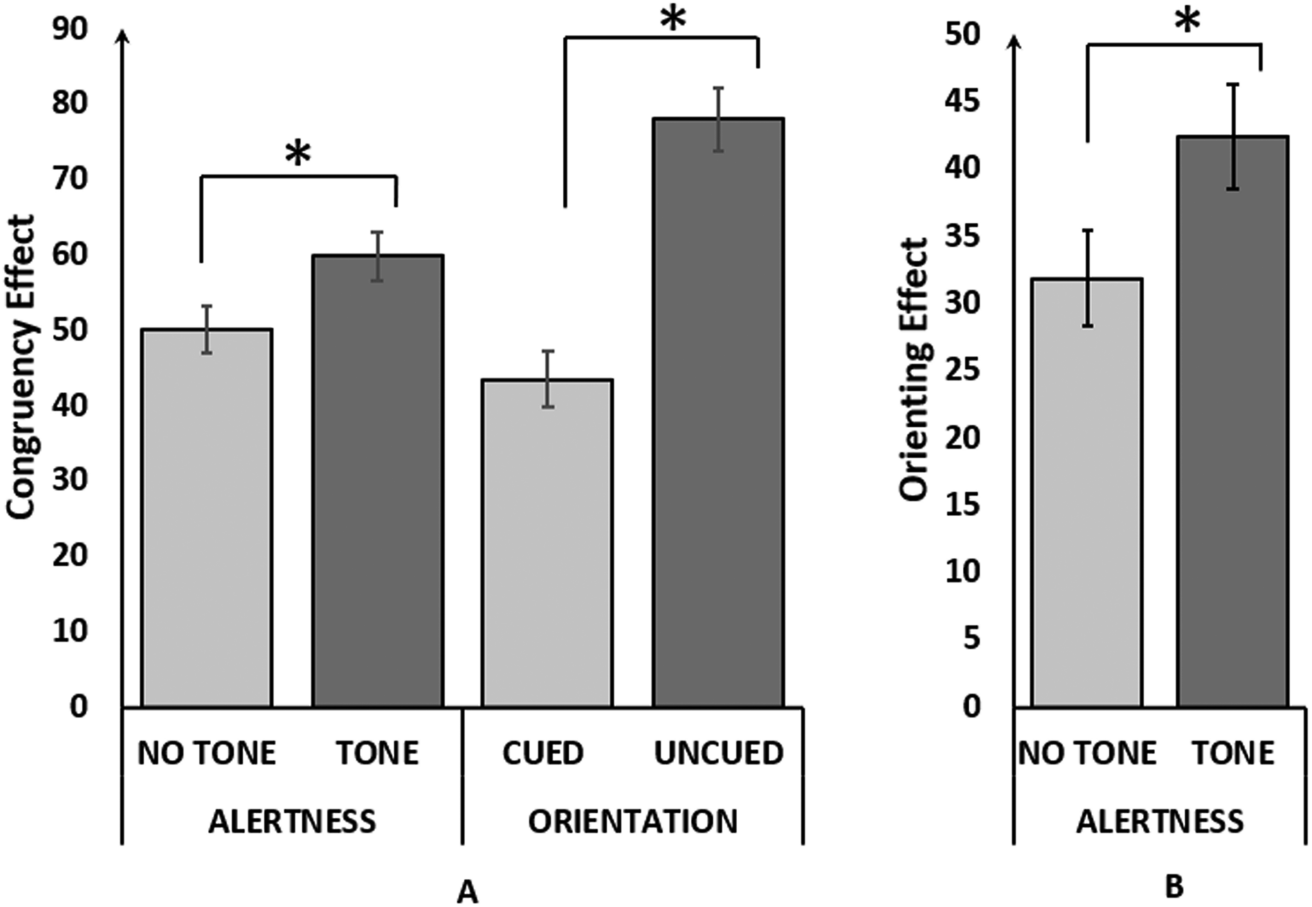
Interactions among the attentional network indices. Interactions between the variables. **(A)** Congruency (mean RT for incongruent trials- mean RT for congruent trials) as a function of cueing (cued trials vs. uncued trials) and alerting (trials with alerting tone vs. trials without alerting tone). **(B)** Cueing (mean RT for uncued trials- mean RT for cued trials) as a function of alerting (trials with an alerting tone vs. trials without an alerting tone). Error bars represent SD and “*” denotes *p* < 0.001.

RTs with extreme values (lower than 200 ms and higher than 1,200 ms) were eliminated and correct trials were tested under the repeated measure ANOVA. Significant main effects were found in the RT for alerting [F (1.77) = 124.04, *p* < 0.001, *η*_p_^2^ = 0.62], orienting [F (2,154) = 123.40, *p* < 0.001, *η*_p_^2^ = 0.62], and executive control [F (1.77) = 584.10, *p* < 0.001, *η*_p_^2^ = 0.88] networks. Responses were faster in tone than no-tone condition, in the valid-cued than invalid cue condition, and in the congruent from incongruent trials presented in Table 1. The interaction between the alerting and executive control networks was significant [F (1.77) = 5.05, *p* < 0.05, *η*_p_^2^ = 0.06]. The congruency effect was significantly lower in the no-tone condition than in the tone condition (Figure 3A). The congruency effect is the differences in the RT of the incongruent and congruent trials representing the executive control network. The orienting×executive control interaction was also found to be significant [F (2,154) = 21.65, *p* < 0.001, *η*_p_^2^ = 0.22]. The congruency effect was larger in invalid than the valid conditions (Figure 3A). A significant interaction was also found between Alerting and Orienting networks [F (2,154) = 17.31, *p* < 0.001, *η*_p_^2^ = 0.18], indicating the orienting effect in the tone condition is larger than no tone condition (Figure 3B). Three-way interactions between the alerting, orienting, and executive control networks were not significant thereby exhibiting a similar pattern with previously published works (30). The mean network scores for the executive control, orienting and alerting networks were 53.9 ms, 28.9 ms, and 45.6 ms respectively. It was not possible to analyze the accuracy (error rate) owing to the lack of variance (0% errors) in some experimental conditions (56).

**Table 1:** Mean RT expressed in ms with the percentage of errors in the parenthesis for each condition**.**

	No alerting tone			Alerting tone		
	No cue	Cued	Uncued	No cue	Cued	Uncued
Congruent	549.8 (2.30)	504.4 (2.06)	517.2 (1.92)	492.9 (2.75)	474.0 (2.2)	501.0 (2.0)
Incongruent	582.7 (3.2)	544.0 (3.2)	594.9 (5.0)	546.7 (4.2)	521.4 (3.8)	579.2 (5.4)

### 3.2 Choice reaction time

The group RT and the accuracy (expressed as% of correct response) were 0.36 ± 0.04 s and 96.9 ± 3.97% respectively.

### 3.3 Physical tests

The group means of the results obtained during different tests are presented in Table 2.

**Table 2:** Physical test results (mean ± SD).

Physical tests	Values
Illinois agility test (s)	16.1 ± 0.78
30 m sprint (s)	4.2 ± 0.29
VO_2_ max (ml/kg/min)	53.1 ± 2.22
Standing broad jump (m)	2.2 ± 0.26

### 3.4 Associations within the physical test parameters

A significant small, positive correlation was found between the CODS ability time and 30 m sprint time (*p* < 0.05). A strong negative correlation was found between the 30 m sprint time and the SBJ (*p* < 0.01) (Table 3). No other correlations were found among the physical tests.

**Table 3:** Correlation coefficients of the physical tests adjusted for BMI and age.

	Standing broad jump (m)	Illinois agility test (s)	30 m sprint (s)	VO_2_ max (ml/kg/min)
Standing broad jump (m)	–			
Illinois agility test (s)	−0.195	–		
30 m sprint (s)	***−0*** *.* ***399*** *	***0.233*** **	–	
VO_2_ max (ml/kg/min)	*0*.*074*	*0.057*	*−0.201*	–

Values presented in italics and plain text indicate Spearman's and Pearson's correlation coefficient respectively; bold value indicates significant correlation. VO_2_ max, maximal oxygen consumption capacity; BMI, body mass index.

* Correlation is significant at the 0.01 level (2-tailed).

** Correlation is significant at the 0.05 level (2-tailed).

### 3.5 Associations between physical test parameters and cognitive test

The executive index of the attentional network has a significantly large, positive correlation with the CODS ability total time (*r* = 0.507, *p* < 0.01) and not with other physical test parameters (Table 4). There was no significant correlation between the physical test parameters and CRT (*r* = −0.011, *p* > 0.05) (Table 4). The attentional network scores were also not mutually correlated indicating their mutually independent nature (Table 5). BMI and age were also not correlated with the attentional network components, CRT, and agility.

**Table 4:** Correlation coefficients among physical and cognitive tests adjusted for BMI and age.

	Standing broad jump (m)	Illinois agility test (s)	30 m sprint (s)	VO_2_ max (ml/kg/min)
Alertness index	*−0* *.* *149*	*−0*.*039*	*0*.*090*	*0*.*080*
Orientation index	−0.051	0.051	*0*.*130*	*0*.*076*
Executive control index	−0.101	.**507^╪^**	*0*.*171*	*−0*.*087*
Choice reaction time	0.040	−0.011	*−0*.*065*	*−0*.*014*

Values presented in italics and plain text indicate Spearman's and Pearson's correlation coefficient respectively; bold value indicates significant correlation.

BMI, body mass index.

* Correlation is significant at the 0.01 level (2-tailed).

**Table 5:** Correlation coefficients among the attentional network scores.

	Alertness index	Orientation index	Executive control index
Alertness index	–		
Orientation index	*0.108*	–	
Executive control index	*−0.116*	0.143	–

Values presented in plain text and italics indicate Pearson's and Spearman's correlation coefficient respectively.

## 4 Discussion

The results of the present study show that CODS ability has a strong, positive, and significant association with the executive control of the attentional network in male young footballers. The positive correlation means that higher the efficiency in the executive attentional network the lesser would be the CODS completion time in the Illinois agility task. For the executive network, a lower network score indicates higher efficiency. This network score is the difference in time taken to identify congruent and incongruent flankers in the ANT-I task. Minimal difference in reaction time explains higher resolving ability or smaller inference control cost, and hence greater efficiency. Taking into consideration the influence of age and BMI on physical activity (57), the association was verified by adjusting their effects. To the best of our knowledge, this is probably the first evidence specifying an association between the executive control of the attentional networks and the CODS ability. The alerting and the orienting networks are not found to be correlated with CODS, indicating that they may not be independently associated with CODS performance. For alerting component, the possible explanation might be that auditory warning signals used to voluntarily engage attention for responding in ANT-I has no additional advantage in a preplanned CODS execution. Alternatively, it could be that the participants already have the level of alertness sufficient for the task. The orienting component of ANT-I is purely exogenous (involuntary) in nature due to the presence of uninformative (invalid) spatial cues and therefore might not found to be associated with endogenously (voluntarily) driven CODS.

CODS ability training is considered to be necessary for conditioning young professional footballers with specific movement patterns and velocities, which replicate the maneuvers of competitive situations of a game. CODS is a closed-skill technique for developing motor proficiency in pattered movement (20). This makes it crucial for the long-term development of football-related skills for enhancing performance. Earlier, CODS ability was considered a subtype of agility due to the speed and direction-change elements. However, the lack of higher-order cognitive skills for reactive response to a target stimulus has made CODS an independent attribute and different from agility (27). We found that the decision-making speed in our CRT task was not correlated with the CODS, which is in agreement with previous report (15). It has been argued whether standard CODS drills can be included to enhance cognitive skills executed in sports environments as they lack decision-making and anticipatory components (20, 26, 27). Our findings have shown that CODS ability is linked with the executive control efficiency of the attentional network, essential for reducing uncertainty from task-irrelevant stimuli and thereby facilitating decision-making.

Attentional control is important in competitive and strategic environment of football (34, 58). It allocates and maintains the focus during sports-specific actions (58). The rationale behind relating the attentional networks with CODS ability is based on the assumption that athletes are efficient in channeling their focus of attention towards a complex motor-skill task to enhance performance. A previous study based on qualitative data has supported that external attention can improve the performance of the change of direction and acceleration task (28). However, this was in the context of directing the focus externally from internally with verbal instructions for better task performance. Our study contributed to further understanding of attentional control on the CODS ability by using a quantitative approach. Our data indicate that enhanced CODS performance might be due to the strong connection with the executive control of the attentional network.

We have chosen the ANT-I task due to the advantage of assessing all three components of attention and their mutual interaction in a single test. ANT-I uses a modified Flanker test to examine the attentional processing in resolving interference either with or without the support of temporal and spatial expectancy regarding target appearance (30). The stimulus expectancy using the alerting and orienting attentional networks is thought to improve response readiness (30). We found that these networks are independent in nature (Table 5) and can have mutual interactions, which replicates the response behavior in the previously published reports (30, 43, 56). ANT-I test has been popular in sports and exercise-related comparative studies examining attentional functioning (34, 56). ANT-I has been used earlier to compare attentional functioning between young elite footballers and non-athletes and with static sports discipline including different age groups and experience (34, 43). These studies have shown higher efficiency of executive control ability in footballers.

We speculated that better motor coordination skills with impulse control could be vital in executing specific movement patterns with high speed in CODS. The executive network is related to monitoring and interference control (32) which may affect impulse control in complex motor-skill tasks. Interference control is one of the core executive functions that selectively allocates attention over task-relevant stimuli and at the same time suppresses the irrelevant stimuli (7). The latter could be physical objects, thoughts, and emotions that might interfere with the goal-directed behavior. Therefore, it can be assumed that athletes exhibiting larger interference costs possibly on these distracting on-field factors are more likely to lose response execution speed in the CODS. This possible explanation is supported by the fact that the brain areas involved in motor planning, execution, and impulse control share identical areas that are associated with executive control network functioning of the ANT-I task (59–61). Another plausible explanation might be that interference control is critical for expert athletes to fix their gaze on target locations of the navigating path during multiple directional changes with high speed to reduce the uncertainty from the interfering objects or obstacles in the environment. Since the safe avoidance strategy during navigation is an important behavior in humans (62). Our assumption is also supported by the fact that cognition in general is an important regulator of gait and balance in older populations (63–65).

Since CODS ability is also a crucial physical component for sports performance, its association with speed, explosive power, and endurance were also tested. It was found that CODS ability is positively correlated with speed and in agreement with the previously published reports (15, 66).

The major strength of the present study is the quantitative estimation of the interference control on motor capabilities that regulate the speed and the multiple changes in the direction of footballers in a predetermined path. Although earlier studies indicated the attentional network functioning in footballers but the association or involvement of specific attentional components were not known. To the best of our knowledge the present study provided first evidence about strong association of the executive control of the attentional network with football-specific CODS.

One of the major limitations of our study is the lack of female athletes that can limit the generalization of our findings. Because, previous literature reported about existing gender differences in perceptual-cognitive abilities in athletes (11, 67, 68). Male athletes are usually better performers than female athletes in some cognitive tasks (67, 68). In future it will be interesting to see whether similar association regarding attentional network components with CODS ability in female athletes. The second limitation of the work is the limited sample size. Although the number of participants was registered based on prior sample size estimation, the association could have been more convincing if tested on a larger population of athletes representing several regional football academies across India. The third limitation may be the accountability of the translation of sports-specific cognitive abilities to computer-based laboratory tasks. The method may not be mimicking the complexities of the game-specific environment, which might have interfered with the degree of mental involvement of the athletes in the tasks. This can be addressed by constructing more realistic and ecologically valid tests capturing cognitive abilities in football-specific setting that requires football-specific motor reactivity (69). The study has taken care of the confounding effects of age and BMI, although it did not consider the other factors as confounding variables such as years of playing experience, quality of sleep, mood, and personality. This could be another limitation of the study.

Future research should take into considerations the inclusion of female athletes, on-field cognitive assessments for strengthening ecological validity. Factors such as training history, sleep quality, and psychological state in future could add valuable insights into the relationship between attentional network functions and CODS in young footballers. It would also be interesting to see whether genetic variation in the young players contribute to attentional network performance in young professional footballers.

## 5 Conclusion & practical implications

It can be concluded from the present study that the efficiency of the executive control of the attentional network in young male footballers may have a positive and significant impact on their CODS ability, whereas it has no association with sprint time, endurance, and explosive power. These findings strengthen a generalized view about the importance cognitive factors on sports-specific motor actions. Our findings provide evidence that CODS ability requires faster mental operations for resolving conflict and build-up movement strategies to move rapidly through a pre-planned multiple-direction path. CODS agility technique is critical for the development of football-related skills and abilities. Therefore, the practical application from the findings can be dual training in ANT-I and CODS ability. This feasible approach can be useful in on-field physical assessments for performance evaluation, talent identification, and screening purposes. The study further encourage coaches and sports science practitioners to integrate dual cognitive-motor training designed to improve executive capabilities (conflict resolution, inhibitory control) in football-specific setting. This integration can provide ecological validity and engaging the network components of attention for giving football-specific motor reactions. The implementation holds promise for the long-term development of cognitive-motor skills, agility and performance optimization in competitive football.

## Data Availability

The raw data supporting the conclusions of this article will be made available by the authors, without undue reservation.
